# Reviews of Books

**Published:** 1924-10

**Authors:** 


### REVIEWS OF BOOKS.

### THE CROSS OF MERCY.

**The Grand Priory of the Order of the Hospital of St. John of Jerusalem in England.** A Short History. By Col. E. J. King, C.M.G., F.S.A., Knight of Grace of the Order. Illustrated. (St. John's Gate, Clerkenwell.)

History provides few more romantic stories than that of the Knights of St. John of Jerusalem. At once monks and soldiers, with the cross on their breasts and the sword in their hands, for centuries they filled a large place in the monastic and military history of Europe. The historian of their Grand Priory of England, in this attractive and well illustrated volume, aptly describes the Order as " the first-born child of the great Crusade," and suggests as its birthday July 15, 1099, when Jerusalem once more fell into Christian hands, and Godfrey of Bouillon knelt for the first time in the Church of the Holy Sepulchre. Within the sacred city he found already in existence a little hospital for Christian pilgrims, which had been created three-quarters of a century earlier by some pious merchants of Amalfi, who had purchased the site of an older hospital established by Charlemagne, but destroyed in 1010 by a fanatical Caliph. It was under the rule of a certain Brother Gerard, and the aid and succour which it gave to the wounded soldiers were so devoted that Godfrey, who, although he had been elected King of Jerusalem, refused to call himself anything more than Defender and Baron of the Holy Sepulchre, endowed it with one of his own manors in Brabant. Other leaders did likewise and the hospital soon became so famous and important and the object of so many benefactions that Brother Gerard and his helpers gave it a regular constitution, took the monastic vows, adopted the Augustinian Rule, and clothed themselves in a black robe with an eight-pointed white cross on the left side near the heart. Such were the beginnings of the great Order which was destined to make so profound an impression upon the world. Within less than a generation the monks had become knights as well, and knights their successors still are, though in a very different sense.

Before long the Order was well established in England, and by the middle of the twelfth century was in possession of its great Priory in Clerkenwell. The long and chequered story of the Hospitallers had now well begun. The disaster of Acre was followed by the Knights' seizure of Rhodes, a virile act which placed them in a highly favourable light as compared with the paralysis that fell upon the Templars when they also lost their footing in Palestine, and helped materially, not only to the final downfall of that Order but to the transfer—only partially effective —of their property to the Hospitallers. A long period of power and influence followed, to be interrupted by the heroic loss of Rhodes in 1522, and the re-establishment of the Order in Malta, which remained its headquarters until it was cast out by Napoleon in 1798. Thus the Knights Hospitallers of St. John of Jerusalem became first Knights of Rhodes and then Knights of Malta. They had not long reigned in their Mediterranean island when, in 1540, they were dissolved in England and their possessions seized, and on the day the Act of Dissolution took effect the Grand Prior, William Weston, died of a broken heart.

There was a brief return to Clerkenwell in Mary's time, but when Elizabeth succeeded her, confiscation again followed, though there was no renewed suppression, and " The Prior and Brethren of the Hospital of St. John of Jerusalem in England " remained a body corporate with perpetual succession as established by Queen Mary. Thus the English *Langue* has never ceased to exist, and in 1888 it was formally revived by a Royal Charter. The rest is fairly familiar. No longer a Sovereign Order, ruling its own territory and granting titles of hereditary nobility, or a mere survival of chivalry as on the Continent, the English Knights of St. John are foremost in works of healing and of mercy, in peace and in war, and still they have their great ophthalmic hospital in Jerusalem, the *urbs beata* in which they took their rise. In Salem they began nearly a thousand years ago, and in Salem they still are to-day, never again, let us hope, to be disturbed.

Col. King has done his work well and modestly. There was need of such a book as this, and he has admirably filled a gap in a great chapter of history.

### CRIMINAL OR INSANE ?

**Crime and Insanity.** By W. C. Sullivan, M.D. (Arnold. 12s. 6d.)

Many of those who have studied Dr. Sullivan's earlier work must have feared that his promotion to an important administrative post, as superintendent of the State criminal lunatic asylum, would deprive us of any further books from his pen. This fear has now been proved to be groundless. We have here an admirable book on a subject of perennial interest and of special importance at the present time. The subject is so handled as to render it intelligible not only to medical and legal readers, but to any educated member of the general public.

Anti-social conduct, so far as society in general is concerned, is the most important practical result of those pathological mental conditions which are grouped together under the title of insanity. A preliminary necessity, therefore, is some consideration as to what we mean by crime. Dr. Sullivan points out the very varied character of the acts and omissions which are included under this name, indicating that the only feature which they have in common is their illegality. He briefly reviews, and rejects, the theory of innate criminality propounded by Lombroso, and carried to extreme lengths by some of the so-called Italian school of criminology, and he holds that crime is to be regarded as a biosocial problem, being, in short, a function of two variables—namely, mental constitution and environment. Of the several ways in which his subject could be treated, Dr. Sullivan selects that of describing the various forms of morbid crime under the headings of the main divisions of insanity. Attention is drawn to the fact that the nature of the emotional tone produced by the mental disease determines, to a large extent, the character of the crime committed. Murder, for example, is uncommon in the exalted phase of manic-depressive insanity, while in the depressed phase of that disease the crime usually possesses no element of malignancy, the melancholic killing those who are dearest to him for the sake of their supposed benefit. The sufferer from systematised delusional insanity usually attacks his supposed persecutors. From the abundant material at his disposal, Dr. Sullivan presents us with a series of admirable clinical pictures. Although all proper care has been taken to obviate general recognition of the cases described, students will be able to identify some as having been of interest at the time of the respective trials, and will find it most helpful to read the histories in the light of the after-trial observations made on the patients.

Each main division of insanity is taken up in turn. Where all is so admirable, it is difficult to particularise. The author's well-known mastery of the subject of alcoholism renders of great value his exposition of the connection between that morbid condition and crime. We may specially commend the chapters on crime connected with epilepsy and with transitory conditions of mental disorder. These are the cases in which controversy usually occurs. Useful rules are given by means of which the simulation which is sometimes attempted may be detected in practice. For instance, loss of memory for the events at the time of the crime is claimed with some frequency. Such loss of memory may be genuine, or it may be feigned. This point is most ably and fairly discussed. " Moral imbecility " is a subject upon which there has been much dispute. Dr. Sullivan recognises the condition, and gives case histories of what he regards as typical examples. He admits, however, that there must be some intellectual defect before the patient can be regarded as a true moral imbecile. It is, therefore, rather difficult to see how these cases differ essentially from those of feeble-mindedness. The criterion of the stealing of articles of little value to the stealer is suggested as one diagnostic point. This would result in moral imbecility being confined to the more wealthy class of society, and it is found, practically, that the diagnosis is reserved for patients of that particular social class. In the estimation of intelligence, Dr. Sullivan admires the original Binet-Simon scale, and does not think that later modifications have improved it. This is not remarkable, inasmuch as he was the first to bring the scale into notice in this country. Very little is said on the new theories of the production of crime by mental conflict and repression; but this view as to the causation of hysteria is accepted.

Probably the section of the book to which most readers will turn first is that in which the vexed question of criminal responsibility is discussed. In his two final chapters Dr. Sullivan gives a most lucid exposition of this complicated subject. He points out that the question is, essentially, a purely legal one, but holds that psychiatrists may properly ask whether the present legal criteria succeed in securing the ends which the law has in view. He urges that the amendment of the McNaughten rulings is a hopeless task, comparable to the sewing of new cloth into an old garment. After indicating the difficulties which would occur in the application of the proposed new test as to the loss of the power of self-control (proposed, be it noted, by a committee of eminent lawyers), he expresses his preference for the law as stated in the French Penal Code. That code enacts that " There is neither crime nor misdemeanour when the accused person was in a state of madness at the time of the act." The practical difficulties which might occur under this rule would not differ essentially from those which are now raised in connection with the McNaughten dicta. And it is probable that most mental specialists who have had experience in this particular direction will share Dr. Sullivan's preference. We may remark that he also adversely criticises the way in which evidence as to an accused person's mental condition is presented to the court, and he favours a system under which a report would be made by a body of independent experts in all cases in which insanity is raised, or is likely to be raised, as a defence.

### MORE LIGHT ON THE COUE METHOD.

**Conscious Auto-Suggestion.** By Emile Coué and Louis Orton. (T. Fisher Unwin. 6s. net.)

There may be many fallacies in M. Coué's teaching, but it is impossible to deny that there is much common sense. Moreover, his books are written in a light and attractive manner and with engaging modesty. He desires to be considered in the light of the doctor's auxiliary :—

> In all cases of serious organic disability, I say to those who seek me out : " Are you receiving medical treatment ? " If they reply, " Yes," I give the advice, " Continue with it then, and practise auto-suggestion also." If they reply, " No," I say, " Consult a doctor then, and follow his treatment as well as using auto-suggestion. You will find that the two treatments help each other." Auto-suggestion is the natural ally of medical precepts. . . . I would say to a doctor, " Give medicines to persons who expect them even if you don't believe in them yourself." And to patients, " Do not insist upon having any medicine ; you may only get coloured water for your pains. Don't run away with the idea that coloured water can do no good however."

Diet, training of children, physical culture—these are a few of the subjects upon which the book touches. " It seems to have been assumed in certain quarters that Couéism is opposed to physical culture." This, quite definitely, is not so, " but we do condemn the attempt to turn persons, independent of the nature of their work, into masses of muscle. . . . Dumb-bell exercise, unless used in conjunction with an alert brain, is definitely harmful." There is a good deal of really fresh information in M. Coué's latest book, and it will certainly find an eager public.

### FOR THE LAYMAN.

**Blood Pressure : Cause, Effect and Remedy.** By Lewellys F. Barker, M.D., LL.D. (Professor Emeritus of Medicine in Johns Hopkins University), and Norman B. Cole, M.D. (Assistant in Clinical Medicine in Johns Hopkins University. (Appleton. 5s.)

This is a book for the layman who, having heard much blood-pressure talk at his club, in the street and elsewhere, wants to know more about it without having to consult text-books bristling with medical terms and immaterial facts. Blood pressure having probably come to stay for some time as a conversational topic among ultra-civilised nations, it is well that a book has been written for popular consumption by authors with a knowledge of facts and the rare gift of presenting them in an easy, conversational manner. Many doctors know the facts, but few can describe them intelligibly ; just as many people can write verse, but few can put it to music.

The authors may be congratulated on having put their verse to music very well. But, like all good artists, they have their foibles. The American seems to be one. He is "all too accustomed to disregard his physical being until symptoms that will not be denied make it plain to him that something is the matter with him." Elsewhere we read that "The majority of Americans eat too much meat by far; there are but relatively few who are of normal weight or heavier who would not be better off physically if they reduced their meat-intake to a half, or even to a third, of the amount they have been accustomed to eat." Let us hope the Americans will take the hint and follow the good old English rule of never—absolutely never—eating bacon and eggs for breakfast, and of limiting this meal to an apple and a piece of dry toast.

The authors append a useful glossary of their terms. Some of their explanations are helpful, as, for example, their definition of capillaries. But when they include "perturbation" in their glossary and define it as "agitation of mind; anxiety," their task is surely supererogatory.

### A REVISED TEXT-BOOK.

**Practical Nursing.** By the late Herbert E. Cuff, M.D., F.R.C.S., and W. T. Gordon Pugh, M.D., B.S. Rewritten and enlarged by Dr. Pugh. 6th Edition. (The Scientific Press, Ltd. 10s. 6d. net.)

The great practical excellence of this book entitles it to a more extensive notice than is usually accorded to a sixth edition. Nearly a quarter of a century ago it made its first appearance as the joint work of Dr. Herbert E. Cuff, a well-known writer for nurses, and Miss Isla Stewart, matron of St. Bartholomew's Hospital, and almost at once leapt into well-deserved popularity. Of the original authors neither is now left. Miss Stewart passed away many years ago, and in 1921 Dr. Cuff lost his life in a brave, though unsuccessful, attempt to save his two daughters from drowning while bathing from the beach. A few days before the tragedy he had just finished making arrangements with his collaborator, Dr. Gordon Pugh, for revising "Practical Nursing," a task which Dr. Pugh has since completed alone with entire success.

We venture to predict a cordial welcome for the volume in its revised form from those whom it is designed to help. The present generation of probationers will be especially attracted by the book, which is now planned to cover all the subjects they have to study in preparation for the State Examinations, both Preliminary and Final, in England and Wales or Scotland. Besides general nursing, hygiene and dietetics, its contents include the curriculum for the supplementary registers for fever and for the nursing of sick children, which can therefore all be studied from a single text-book. The subjects for examination are further detailed in a special appendix, in which is indicated, chapter by chapter, the items specified in the several syllabuses, charts and schedules. With this assistance, therefore, the student will learn how to read the book to the best advantage and how to test her newly-acquired knowledge as she goes along. The sister-tutor also should benefit from this help in arranging her classes. Revision has been extensive and thorough, and there are numerous and excellent illustrations.

### THE SEARCH FOR A CANCER CURE.

**Cancer Research at the Middlesex Hospital, 1900-1924: Retrospect and Prospect.** Compiled by members of the Staff of the Hospital and Medical School. Edited by W. Sampson Handley, M.S., F.R.C.S. (John Murray for the Middlesex Hospital Press. Price 3s. 6d. net.)

For many years the Middlesex Hospital and its associated departments have been a centre for the treatment of cancer patients and for cancer research. In 1792 wards were first provided for sufferers from this disease, where they could remain until "relieved by art or released by death." In 1857 a "Report on Cancer" was issued by the surgical staff, and the policy of paying particular attention to the cancer problem was steadily pursued, until, in 1900, the special cancer research laboratories were inaugurated. From this point still further development occurred, facilities for both clinical and laboratory research being systematically increased. Special wards, containing some ninety beds, are now set aside solely for cancer cases, which are at the same time admitted to the general wards. In 1913 the Board of the Hospital realised that the new developments of the radiation treatment of cancer and the care of their stock of radium demanded the attention of a whole-time physicist, and this appointment, the first of its kind in the country, was duly made.

So might one trace from the pages of this volume many attractive details in the growth of this important unit in cancer research. But more essentially useful is the clear explanation provided in the book as to what these particular investigations imply, of the actual nature of the work undertaken, and of the multitudinous difficulties which it is often impossible for the uninitiated to realise. As a record of work done by one hospital department the whole production is excellent and remarkably stimulating to those who have the success of its efforts at heart. Also, as a point of public interest, proof is afforded that the laboratory researches have undoubtedly led to great and definite improvements in the practical treatment of cancer.

### MENTAL ABNORMALITIES.

**Mental Diseases.** By R. H. Cole, M.D., F.R.C.P. (The University of London Press. 15s. net.)

We welcome the third edition of this well-known text-book, which was originally published in 1913. The student will find the volume to contain all that he requires for his final examination; and the general practitioner will obtain most useful assistance in the diagnosis of what are, perhaps, the most difficult cases with which he has to deal. The present edition has been brought thoroughly up-to-date, the malaria treatment for general paralysis being referred to. The descriptions of the varieties of mental abnormality are admirably clear; differential diagnosis is carefully detailed, and we would mention with special approbation the chapter dealing with the elements of prognosis.

The book rightly starts with a brief statement of the philosophical problems involved. Failure to form definite opinions on these vital questions is at the root of much confusion. Dr. Cole adopts a monistic position, while apparently recognising, with McDougall, that this view does not necessarily exclude animism. Without explicitly stating the fact, the book seems to assume the position of scientific determinism. But it seems somewhat misleading to attempt to justify punishment on the ground that the lawbreaker's malorganisation "has proceeded from failure to stimulate his higher nature." The real justification for punishment, on deterministic principles, is that the possibility of retribution introduces a new factor into the case. The potential lawbreaker is not in the same circumstances as he would have been had the possibility of punishment been non-existent. As regards the abstruse question of criminal "responsibility," Dr. Cole, while he indicates the gaps in the "McNaughten dicta," seems to think that, on the whole, no substantial injustice is done to the insane in criminal trials. With this view most people would probably agree.

On the Freudian theory Dr. Cole writes with most eminent fairness. The main outlines of this most important subject are briefly but accurately stated. The immense value of Freud's hypothesis is fully recognised, both as regards its therapeutic applications and as regards the light which it throws upon the mechanism of production of the neuroses and psychoses. The subject of sex abnormalities is one upon which the general practitioner needs much guidance; and this is the weakest part of the book. The illustrations are excellent. The book is splendidly printed, and there is a sufficient index.

### MISCELLANEOUS NOTICES.

### For the Masseur.

Massage of the Head, Face and Neck. By Olive F. Sands, Masseuse to Savernake Hospital, Marlborough. Pocket Guide Series. (Scientific Press. 1s. 6d.)—This useful little book tells simply and clearly how the masseur or masseuse may best achieve the results expected by the doctor. There is a short chapter on "Massage to Improve Personal Appearance," which, though not indispensable to the recovery of the patient, has been found to have undoubted stimulative merit, particularly in the case of female patients.

### The Practice of Medicine.

Wheeler's Handbook of Medicine. By William R. Jack, B.Sc., M.D. Seventh Edition. (E. and S. Livingstone. 12s. 6d. net.)—Numerous additions and alterations have been made in this, the seventh, edition of this popular text-book. These include the insulin treatment of diabetes, bacillus coli infections, encephalitis lethargica, and pneumococcus infections among others. The whole work has undergone careful revision and is up to date in every respect. Concisely written and easy to read, it is probably the best one-volume text-book of medicine to place in the hands of senior students and junior practitioners.

### Renal Disease.

Modern Methods in the Diagnosis and Treatment of Renal Disease. By Hugh Maclean, M.D., D.Sc. 2nd Ed., revised and enlarged. Illustrated. (W. Constable.)—As the author explains, no results of much clinical importance have, in this respect, been described since the first edition was published in 1921. But further general experience has allowed some expansions, and in the present edition a fuller discussion of the indications afforded by high blood-urea concentration is given. This work, already well known, amply meets the needs of medical men.

### For Insurance Practitioners.

Medical Insurance Practice. By R. W. Harris and Leonard Shoeten Sack. 2nd Edition. (Scientific Press. 7s. 6d. net.)—The writers of this book, one a late assistant secretary in the Ministry of Health, the other a barrister, are well equipped by their professions for the authorship of such a work. It is addressed "to the busy general practitioner," for whom it sets out clearly and as briefly as is consistent with accuracy his obligations to his insured patients, how he can enter the service, and how he will be remunerated for his work. Doctors or laymen concerned with Health Insurance work will find it extremely valuable as a book of reference and of explanation of the necessarily complex workings of so large an organisation as National Health Insurance. In connection, too, with the Resolution of the General Medical Council (May, 1922) that instruction should be given on the duties which devolve upon practitioners in their relation to the State, the book may warmly be commended to the attention of those entrusted with the courses of study in Medical Schools.

### Dr. Rollier's Sun Cure.

The Healer: How to Fight against Tuberculosis. By Dr. Auguste Rollier. (People's League of Health, 12, Stratford Place, W. 1. 1s.)—There are few people to-day who have not heard of Dr. Rollier's sun-cure method for the treatment of tuberculosis and its wonderful results, but there must be many anxious to know just how the treatment is carried out. From "The Healer" we may learn how tuberculosis can be prevented in the young and how even advanced cases can generally be cured. It shows that at the clinics at Leysin, while bodies are being cured, minds are being educated, for it is one of Dr. Rollier's theories that, as Dr. Vigné has said: "Boredom, although not pathologically classified, is a very serious disease. By destroying the will, boredom is the forerunner of failure, for in tuberculosis, more than in any other disease, success is often a question of potential nervous energy, and one must 'will' unceasingly to be cured in order to become so."

### Practical X-Ray Work.

A Manual of Practical X-ray Work. By John Muir, B.Sc., M.B., in collaboration with Sir Archibald Reid and F. J. Harlow. (Heinemann. 31s. 6d. net.)—Since this work was first published in 1908 radiology, as an auxiliary to medicine and surgery, has progressed with such remarkable speed that extensive revisions and enlargement have been necessary. In the present edition—the third—every branch of radiological work is dealt with. From the doctor's point of view it will be satisfactory that the sections on diagnosis occupy a proportionately greater space than before. The scope of the revision has, in fact, been guided to some extent by the inclusion of radiology as a necessary subject of study in the curriculum of all medical students seeking British qualifications. For this simple purpose the book may be a little advanced, as sufficient detail has been included to furnish a comprehensive introduction to study for the special radiological diplomas. There is no doubt, however, that to give practising physicians and surgeons an intelligent conception of the possibilities and limitations of radiological assistance the new "Arthur & Muir" should amply serve its purpose.

### Examination Aids.

Materia Medica and Pharmacology for Nurses. By Gwendolin Hindes, M.Sc. (The Scientific Press, Ltd.)—Though this book is not large, a great deal of information is packed away within its 158 pages, and it will take many hours of solid reading and steady application before the knowledge it contains can be thoroughly assimilated even by a persevering student. The little volume belongs to the "Nurses' Examination Aid Series," which is being published by the Scientific Press to meet an increasing and steady demand for helps of this kind now that State examinations are the order of the day. The author of "Materia Medica" is well qualified for the work she has so ably carried out. Herself a member of the Pharmaceutical Society, and also pharmacist at the York County Hospital, she has made a course of lectures given by her to the nurses in training at that institution the basis of her book, and has approached the subject from the point of view of one accustomed to teach those who are tackling, perhaps for the first time, a study unfamiliar, and to them, abstruse. Excluding as far as possible the kindred but difficult subjects of botany and chemistry, she has therefore aimed at focusing the learner's attention more directly on the nature and action of the drugs themselves on the body, which, after all, is the practical end of a nurse's studies in this direction.

